# A systematic review and meta-analysis of bone metabolism in prostate adenocarcinoma

**DOI:** 10.1186/1471-2490-10-9

**Published:** 2010-05-19

**Authors:** Ary Serpa Neto, Marcos Tobias-Machado, Marcos AP Esteves, Marília D Senra, Marcelo L Wroclawski, Fernando LA Fonseca, Rodolfo B dos Reis, Antônio CL Pompeo, Auro Del Giglio

**Affiliations:** 1 Urologic Oncology Division; Dept. of Urology; ABC Medical School (FMABC); Santo André, Brazil; 2 Research Institute; Albert Einstein Jewish Hospital (IEP-HIAE); São Paulo, Brazil; 3 Oncology Division; Dept. of Clinical Oncology and Haematology; ABC Medical School (FMABC); Santo André, Brazil; 4 Dept. of Urology; USP Medicine School (FMUSP-RP); Ribeirão Preto, Brazil

## Abstract

**Background**

Osteoporosis could be associated with the hormone therapy for metastatic prostate carcinoma (PCa) and with PCa *per se*. The objective of this review is to determine the incidence of bone loss and osteoporosis in patients with PCa who are or are not treated with hormone therapy (ADT).

**Methods**

The Medline, Embase, Cancerlit, and American Society of Clinical Oncology Abstract databases were searched for published studies on prostate cancer and bone metabolism. The outcomes assessed were: fracture, osteoporosis and osteopenia.

**Results**

Thirty-two articles (116,911 participants) were included in the meta-analysis. PCa patients under ADT had a higher risk of osteoporosis (RR, 1.30; *p *< 0.00001) and a higher risk of fractures (RR, 1.17; *p *< 0.00001) as compared to patients not under ADT. The total bone mineral density was lower in patients under ADT when compared with patients not under ADT (*p *= 0.031) but it was similar to bone mineral density found in healthy controls (*p *= 0.895). The time of androgen deprivation therapy correlated negatively with lumbar spine and total hip bone mineral density (Spearman's rho = -0.490 and -0.773; *p *= 0.028 and 0.001, respectively) and with total hip *t *score (Spearman's rho = -0.900; *p *= 0.037).

**Conclusion**

We found consistent evidence that the use of androgen deprivation therapy in patients with PCa reduces bone mineral density, increasing the risk of fractures in these patients.

## Background

Prostate cancer (PCa) is the most common cancer in men in many Western countries and is the second leading cause of cancer death in men [1]. PCa is characterized by its propensity for bone metastases which occur in more than 80% of patients with advanced prostate cancer [2,3]. Typical metastasis sites include the spine, pelvis and rib cage[4]. The median survival time of patients with PCa is approximately three years after the development of bone metastases, and during this period, patients are at risk of pain, hypercalcaemia, fracture and spinal cord compression [5].

Another feature of patients with PCa is bone loss and, in a more advanced period, osteoporosis. Antihormonal therapy used to inhibit the disease progression or prevent its recurrence can lead to changes in bone metabolism, resulting in the loss of bone mineral density (BMD) since this therapy depletes circulating levels of oestrogens and androgens that maintain bone mass through the suppression of bone reabsorption and promotion of bone formation [6]. These pathological changes are known as cancer treatment-induced bone loss (CTIBL). However, patients with prostate cancer typically have low bone mineral density (BMD) even before receiving hormone therapy as a result of age, underlying disease, or other co-morbidities [7].

Bone mass loss and osteoporosis may cause an increased risk of fractures due to a reduction in bone volume and microarchitectural deterioration. The WHO expert committee defines osteoporosis as a hip bone mineral density level (dual x-ray absorptiometry) of more than 2.5 SD below the mean for young, white, adult men (with a t-score of at least - 2.5 SD) in men age 65 years and older and in men from 50 to 64 years of age if other risk factors for fracture are presented [8]. The most significant complications of osteoporosis are fractures of the hip, forearm, and vertebrae. The occurrence of fractures significantly correlate with shorter survival in men with prostate cancer. When fracture history was evaluated, the median overall survival time was 39 months longer in men without a history of skeletal fracture. Therefore, a better understanding of the magnitude and prevalence of bone loss in these patients is critical [9,10].

The objective of this review is to determine the incidence of bone loss and osteoporosis in patients with PCa who are or are not treated with hormone therapy.

## Methods

### Search methods for identification of studies

Studies were identified through a computerized search of Medline (1966-2009), Cancerlit (1966-2009), and Embase (1990-2009), databases using the following as search query: "prostate cancer and (osteoporosis or bone mineral density)". A computerized search of the Proceedings of the Annual Meetings of the American Society of Clinical Oncology (ASCO) held between 1998 and 2008 was also performed to identify relevant studies published in abstract form. Lastly, all review articles and all cross-referenced studies from retrieved articles were screened for pertinent information.

### Selection of studies

The meta-analysis was limited to studies that involved with the relationship of prostate cancer and/or hormone therapy with osteoporosis and in any language. For the incidence analysis all studies that report these rates were included (Table 1). Studies were excluded if fracture outcome or BMD data were not provided, or if they included patients with other bone or mineral disorders. When we found duplicate reports of the same study in preliminary abstracts and articles, we analyzed the data from the most complete data set.

**Table 1:** Characteristics of studies included in the meta-analysis

		Bone Metabolism Assessment		
			
Source	Total No. Of Patients	DEXA	Biomarkers of Turnover	Type of Patients
Agarwall et al,[11] 2004	50	Yes	None	PCa before and after ADT
Ahlborg et al,[12] 2008	754	Yes	None	PCa, PCa with ADT and controls
Bernat et al,[13] 2005	18	Yes	None	PCa with ADT
Berruti et al,[14] 2005	200	None	Yes	PCa with ADT
Bruder et al,[15] 2006	89	Yes	Yes	PCa with ADT
Chen et al,[16] 2001	109	Yes	None	PCa with ADT and controls
Conde et al,[17] 2004	34	Yes	None	PCa
Daniell et al,[18] 2000	54	Yes	None	PCa, PCa with ADT and controls
Diamond et al,[19] 2004	87	Yes	Yes	PCa with ADT
Galvão et al,[20] 2008	72	Yes	Yes	PCa before and after ADT
Greenspan et al,[21] 2005	195	Yes	Yes	PCa, PCa with ADT and controls
Hatano et al,[22] 2000	218	Yes	Yes	PCa with ADT
Higano et al,[23] 2004	17	Yes	None	PCa before and after ADT
Kiratli et al,[24] 2001	36	Yes	None	PCa, PCa with ADT and controls
Lee et al,[25] 2005	65	Yes	None	PCa before and after ADT
Maillefert et al,[26] 1999	12	Yes	Yes	PCa before and after ADT
Malcolm et al,[27] 2007	395	Yes	None	PCa with ADT
Miyaji et al,[28] 2004	27	Yes	Yes	PCa before and after ADT
Morote et al,[29] 2007	390	Yes	None	PCa before and after ADT
Oefelein et al,[9] 2002	195	Yes	None	PCa with ADT
Panju et al,[30] 2008	66	Yes	None	PCa with ADT
Ryan et al,[31] 2007	120	Yes	None	PCa with ADT
Shahinian et al,[32] 2005	50,613	Yes	None	PCa and PCa with ADT
Smith et al,[33] 2001	41	Yes	Yes	PCa
Smith et al,[34] 2005	11,661	Yes	None	PCa and PCa with ADT
Smith et al,[35] 2006	12,120	Yes	None	PCa and PCa with ADT
Spanjol et al,[36] 2008	398	Yes	None	PCa with ADT
Stoch et al,[37] 2001	257	Yes	Yes	PCa, PCa with ADT and controls
Townsend et al,[38] 1997	224	Yes	None	PCa with ADT
Wei et al,[39] 1999	32	Yes	None	PCa before and after ADT
Yamada et al,[40] 2007	204	Yes	Yes	PCa with ADT and controls
Alibhai et al,[41] 2009	38,158	None	None	PCa with ADT and controls

DEXA: Dual energy X-ray absorptiometry; PCa: Prostate cancer; ADT: Androgen deprivation therapy

### Data extraction and statistical management

Data were independently extracted from each report by M.A.P.E, A.S.N and M.D.S, using a data recording form developed for this purpose. After extraction, data were reviewed and compared by A.S.N. Instances of disagreement between the two other data extractors were resolved by consensus among the investigators. Whenever needed, we obtained additional informations about a specific study by directly questioning the principal investigator.

For the fracture analysis, we computed a pooled estimate of the risk ratios (RRs) of each study using a fixed effect model according to Mantel and Haenszel and graphically represented these results in forest plot graphs. The homogeneity assumption was verified with a χ^2 ^test, using a *df *equal to the number of analyzed studies minus one. An estimate of the potential publication bias was performed by plotting the single study RR on a log-scale against the respective standard error (SE) creating a funnel plot. We used bivariate correlations with the Spearman's rho coefficient to assess the relationship between ADT time and bone mineral density.

For all analyses, *p *values < 0.05 were considered significant. For publication bias, *p *values < 0.1 were considered significant.

## Results

### Literature search

**Figure 1:**
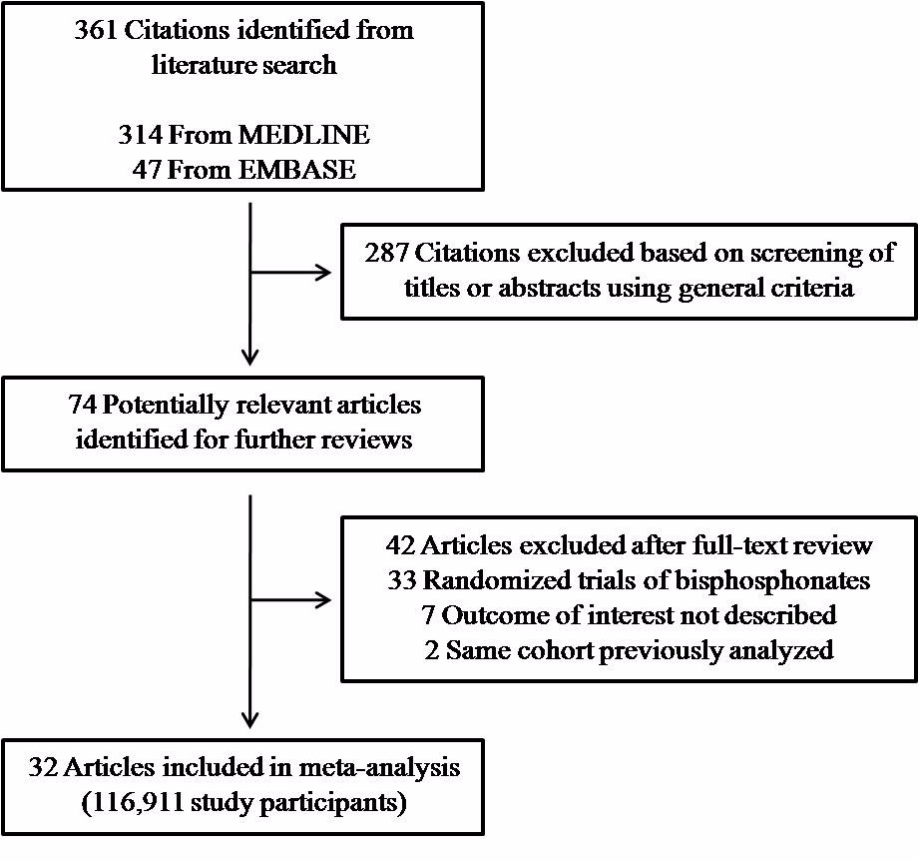
**Flowchart of the meta-analysis**.

The search strategy retrieved 361 unique citations: 314 from MEDLINE and 47 from EMBASE. Of these, 287 were excluded after the first screening, which was based on abstracts or titles, leaving 74 articles for full-text review (Figure 1). During this review, 42 articles were excluded for the following reasons: they involved randomized controlled trials with bisphosphonate therapy (*n *= 33); the same cohort was previously analyzed (*n *= 2); or the bone mineral density, *t *or *z*-scores or osteoporosis/osteopenia rate was not shown (*n *= 7). Finally, 32 articles (116,911 participants) were included in the meta-analysis.

### Study characteristics

The characteristics of the 32 selected studies are shown in Table 1[11-41]. With two exceptions [14,41], all studies reported bone mineral density values as assessed by dual energy X-ray absorptiometry. Eleven studies reported biomarkers of bone turnover biomarkers, including alkaline phosphatase and cross-linked N-telopeptide of type I collagen (NTx). Twelve studies evaluated patients with prostate cancer (PCa) under androgen deprivation therapy (ADT); eight evaluated the same cohort of patients with PCa before and after the ADT; five evaluated patients with PCa, patients with PCa under ADT and healthy controls; three evaluated patients with PCa and patients with PCa under ADT; two evaluated patients with PCa under ADT and healthy controls; and two evaluated only patients with PCa.

The selected studies were published between 1997 and 2009, and the number of participants per study ranged from 12 to 50,613, for a total of 116,911 participants. At the baseline, the number of participants with PCa was 70,684, the number of participants with PCa under ADT was 45,161, and the number of healthy controls was 1,066. The mean age of the participants varied from 66 to 79 years (72.33 ± 3.12 years), and the mean time of ADT in the patients treated with this therapy varied from 2.9 to 120 months (36.98 ± 31.29 months). The risk ratios for osteoporosis, osteopenia and fractures were determined for seven, two and five studies respectively.

### General characteristics of the patients

**Figure 2:**
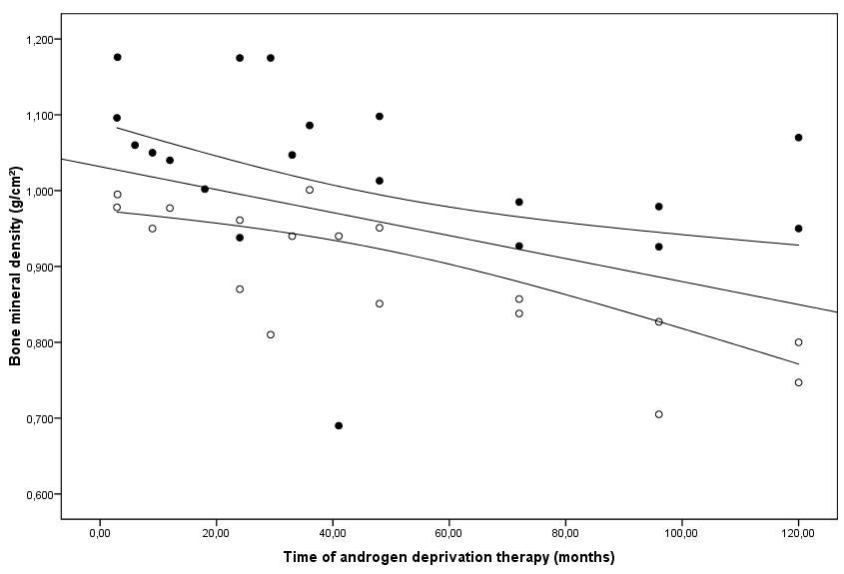
**Scatter plot of lumbar spine (*black circles*) and total hip (*white circles*) BMD and ADT time**.

The general characteristics of the participants are described in Table 2. Patient age was similar in all groups. The total bone mineral density was lower in patients under ADT when compared with patients without ADT (*p *= 0.031), but it was similar to those found in healthy controls (*p *= 0.895). The total bone mineral density of the total hip was lower in patients under ADT when compared with patients without ADT (*p *= 0.002), but it was similar to those values found in healthy controls (*p *= 0.211). The *z *score was similar in all groups, and the *t *score of the lumbar spine and total hip was lower in patients under ADT when compared with patients with PCa without ADT (*p *= 0.031 and *p *= 0.021, respectively). The time of androgen deprivation therapy correlated negatively with lumbar spine and total hip BMD (Spearman's rho = -0.490 and -0.773; *p *= 0.028 and 0.001, respectively) and with total hip *t *score (Spearman's rho = -0.900; *p *= 0.037). (Figure 2)

**Table 2:** General characteristics of the participants

	PCa and ADT (*n *= 26,082)	p*	PCa w/ADT (*n *= 51,605)	p**	Controls (*n *= 1,066)	p***
Age (years)	72.3 ± 3.12	> 0.05	70.2 ± 2.81	> 0.05	70.3 ± 3.30	> 0.05
ADT time (months)	36.9 ± 31.2	---	---	---	---	---
Total BMD (g/cm^2^)	0.90 ± 0.34	0.031	1.07 ± 0.11	0.760	0.96 ± 0.20	0.895
LS BMD (g/cm^2^)	1.02 ± 0.10	0.083	1.10 ± 0.13	0.806	1.05 ± 0.18	0.868
TH BMD (g/cm^2^)	0.89 ± 0.08	0.002	1.01 0.08	0.823	0.97 ± 0.03	0.211
*t *score (Total)	-1.30 ± 1.10	0.282	-0.26 ± 1.14	---	---	---
*t *score (LS)	-0.27 ± 1.21	0.031	0.25 ± 0.07	---	---	---
*t *score (TH)	-0.94 ± 0.24	0.021	-0.55 ± 0.07	---	---	---
*z *score (Total)	-0.30 ± 0.69	0.164	0.54 ± 0.15	---	---	---
*z *score (LS)	-0.27 ± 1.31	---	---	---	---	---
*z *score (TH)	-0.33 ± 0,65	0.555	0.05 ± 0.35	---	---	---
Osteoporosis (%)	5.30	< 0.001	2.89	< 0.001	10.3	< 0.001
Osteopenia (%)	1.01	< 0.001	0.15	< 0.001	1.4	0.278
Fracture (%)	17.56	< 0.001	15.62	< 0.001	1.5	< 0.001
Vertebral fracture (%)	2.96	< 0.001	1.90	---	---	---
Superior member fracture (%)	4.45	< 0.001	2.47	---	---	---
Inferior member fracture (%)	9.77	< 0.001	7.38	---	---	---

LS: Lumbar spine; TH: Total hip

*: PCa and ADT *vs *PCa w/ADT

**: PCa w/ADT *vs *Controls

***: Controls *vs *PCa and ADT

The incidence of osteoporosis was higher in patients under ADT when compared with patients with PCa without ADT (*p *< 0.001), but it was lower when compared with the healthy controls (*p *< 0.001). However, patients under ADT had a higher number of fractures when compared with patients with PCa and healthy controls (*p *< 0.001 for both comparisons).

### Risk of osteoporosis and fracture

**Figure 3:**
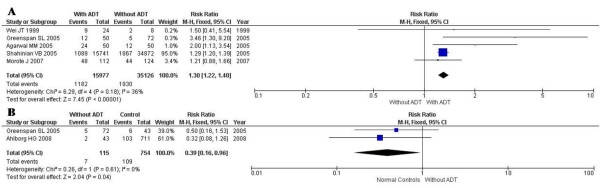
**Adjusted risk ratios**. A, Adjusted risk ratio of osteoporosis for patients under ADT as compared to patients not under ADT. B, Adjusted risk ratio of osteoporosis for patients with PCa as compared to healthy controls.

Among the five selected studies that analyzed patients under ADT and patients with PCa only, all found an association between androgen deprivation therapy and an increased risk of osteoporosis. Patients with PCa under androgen deprivation therapy had an increased risk of developing osteoporosis as compared to patients with PCa who were not under ADT, with a pooled risk ratio (RR) of 1.30 (95% CI, 1.22 - 1.40) (Figure 3A).

Of the two studies that analyzed patients with PCa not under ADT and healthy controls, neither found an association between PCa and an increased risk of osteoporosis. Patients with PCa without ADT had a reduced risk of developing osteoporosis as compared to healthy controls, with a pooled RR of 0.39 (95% CI, 0.16 - 0.96) (Figure 3B). Patients under ADT had a higher risk of developing osteoporosis when compared to healthy controls (RR, 2.26; 95% CI, 1.00 - 5.09).

**Figure 4:**
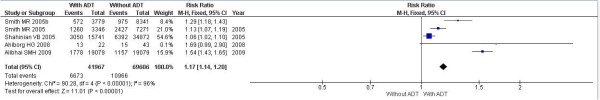
**Adjusted risk ratio of fractures for patients under ADT as compared to patients not under ADT**.

Among the five selected studies that analyzed patients with PCa under ADT and patients with PCa without ADT, all found an association between androgen deprivation therapy and an increased risk of fractures, with a pooled RR of 1.17 (95% CI, 1.14 - 1.20), but with significant heterogeneity of RRs across studies (*p *< 0.0001; I^2^, 96%). These measurements of heterogeneity were likely a result of the extremely large overall number of participants in our analysis (111,573 participants). The point estimates of the RRs were consistently greater than one in all studies. (Figure 4)

To explore the study heterogeneity, we performed stratified analyses across a number of key study characteristics and clinical factors (Table 3). The finding that patients under ADT had an increased fracture risk was consistently found in all of the stratified analyses. For example, when stratified by the type of the fracture, patients under ADT seemed to have a higher risk of lumbar spine fracture than of hip/femur fracture.

**Table 3:** Stratified analyses of pooled relative risk of fractures for patients under androgen deprivation therapy

Stratified Analysis	Patients	Pooled RR (95% CI)	Heterogeneity
Incidence of fractures as the primary outcome			
Yes	111,573	1.17 (1.14 - 1.20)	0.0001
No	---	---	---
Type of outcome measure			
Self-reported	50,613	1.06 (1.02 - 1.10)	Not applicable
Ambulatorial	22,802	1.18 (1.12 - 1.24)	0.02
Bone metastases in the sample			
Yes	50,678	1.06 (1.02 - 1.10) 1.18	0.09
No	22,737	(1.12 - 1.23)	0.01
Mean follow-up, y			
≥ 5	61,295	1.08 (1.04 - 1.11)	0.04
< 5	12,120	1.29 (1.18 - 1.43)	Not applicable
Type of fracture			
Lumbar spine fracture	74,394	1.33 (1.22 - 1.45)	0.93
Inferior member fracture	74,394	1.15 (1.10 - 1.20)	0.001

### Publication bias

**Figure 5:**
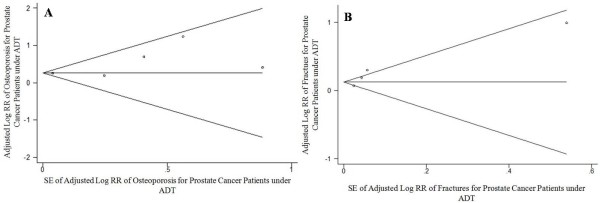
**Funnel plots**. A, Begg funnel plot with pseudo 95% CIs for osteoporosis analysis of patients under ADT (Figure 2A). B, Begg funnel plot with pseudo 95% CIs for fracture analysis of patients under ADT (Figure 3).

The visual inspection of the Begg funnel plot that is related to figure 3A did not revealed asymmetry (*p *= 0.219) (Figure 5A). This finding is consistent with a small possibility of publication bias, as confirmed by the Begg test (z = 1.47; *p *= 0.142). The visual inspection of the Begg funnel plot that is related to figure 3B did not revealed asymmetry (*p *= 0.193) (Figure 5B). This results excludes the possibility of publication bias, as confirmed by the Begg test (z = 0.68; *p *= 0.497).

## Discussion

An extensive body of literature reports on the association between androgen deprivation therapy and the incidence of osteoporosis. All the five studies that we identified, and that met the inclusion criteria, indicated a positive association between ADT and osteoporosis. In relation to fractures, all four of the identified studies indicated a positive association between ADT and the incidence of fractures. Furthermore, the association persisted and remained statistically significant across a number of stratified analyses that explored clinical and study quality factors.

Observational primary studies usually cannot prove causality. However, the studies in this review presented an appropriate temporal relationship; the androgen deprivation therapy and the diagnosis of prostate cancer diagnosis preceded the incidence of osteoporosis and fractures in all of the studies. Furthermore, androgen deprivation therapy depletes the circulating levels of oestrogens and androgens that maintain bone mass through suppression of bone reabsorption and promotion of bone formation [6]. These facts impart biological plausibility to our findings on the association between PCa and ADT with osteoporosis and fractures as shown by the forests plots (Figure 3A, 3B and 4).

The lack of adjustment for the presence of metastasis (only one study adjusted for this factor), calcium ingestion (no studies) and genetic predisposition (no studies) must be considered as a limitation of our study. The presence of metastasis, a low calcium ingestion and patients with history of osteoporosis in the family had a higher risk of osteopenia, osteoporosis and fractures.

It is estimated that two million men are affected by osteoporosis in the United States. Although men experience a gradual age-related loss of BMD of 7 to 12% per decade beginning at age 30, primary male osteoporosis is not common [42]. Most men who have clinically significant osteoporosis are older than 70 years of age and have risk factors that contribute to decreased bone mineralization, such as hypogonadism, thyroid and parathyroid disorders, glucocorticoid excess, alcoholism, osteomalacia, and malignancy [43]. The relationship between decreased BMD and ADT is well established. Androgen suppression reduces BMD approximately 3% to 7% per year [44]. Recent reports have demonstrated that men who have prostate cancer and are receiving ADT have BMD measurements from 6.5% to 17.3% lower than men who are not treated with ADT [7]. One study reported that spinal and femoral BMD values were 1.7% and 5.2% less after two years and 14% and 28% less after 10 years of ADT, respectively, as compared to age-matched control subjects [24].

Although some studies defend the idea that PCa patients (without ADT) have lower levels of BMD and higher rates of osteoporosis, we were not able to verify this finding in our study. In fact, patients with prostate cancer showed similar levels of BMD and lower rates of osteoporosis when compared with healthy controls. Assessed individually, three studies showed similar levels of BMD between PCa patients and healthy controls [21,24,37], one showed higher levels for the PCa group [12] and one showed lower levels [18]. With respect to osteoporosis, all studies showed lower rates in the PCa group as compared to healthy controls [12,21]. One potential explanation for these findings is the highly debated association of a high endogenous androgen levels with the risk of prostate cancer, which may potentially explain the higher BMD seen in patients with PCa before they receive ADT as compared to normal men [45]. We were unable to evaluate this hypothesis in our study because only two of the evaluated studies had data on testosterone levels. Nevertheless, the prevalence of osteoporosis in man with PCa cannot be neglected.

Wei *et al *[39] found that 63% of patients who had hormone-naive prostate cancer had osteopenia or osteoporosis. In a larger study, Smith *et al *[33] demonstrated that 34% prostate cancer patients without exposure to ADT had dual-energy x-ray absorptiometry (DEXA) criteria for osteopenia or osteoporosis. Conde *et al *[17] reported a high prevalence of osteopenia (73.5%) and osteoporosis (17.6%) in 34 men who had non metastatic, hormone-naive prostate cancer. We could see that prostate cancer and ADT are strongly associated with bone metabolism modifications and, in these studies, advanced age, lower body mass index, and elevated prostate specific antigen levels correlated significantly with decreased BMD.

Fractures are associated with substantial morbidity and mortality. Men who experience hip fractures suffer greater impairment and have a higher rate of fracture-related mortality than women [46]. Approximately 20% to 30% of hip fractures occur in men, and 50% to 60% of men die within one year of the fracture [47]. The detrimental association between fracture and mortality extends to men who have prostate cancer. Oefelein and colleagues identified a negative association between skeletal fracture and overall survival in 195 prostate cancer patients treated with chronic ADT [9].

The bone mass of a normal adult is the outcome of a dynamic equilibrium between bone formation and bone resorption. The latter step is regulated by a family of proteins that include receptor activator of nuclear factor k-Β (RANK), RANK ligand (RANKL) and osteoprotegerin (OPG). Binding of RANKL to RANK on the surfaces of osteoclast precursors will trigger maturation, activation, and prolonged survival of these cells. Thus, RANKL promotes bone resorption. Vitamin D, parathyroid hormone, tumour necrosis factor-α (TNF-α), activated T-cells, and glucocorticoid therapy all increase this ratio, promoting bone resorption. Estrogen deficiency states produce osteoporosis because normal levels of 17β-estradiol inhibit RANKL production and stimulate OPG. Testosterone stimulates osteoblasts, inhibits the apoptosis of both osteoblasts and osteoclasts, and is a precursor of estrogen via aromatization; its net effect is to stimulate bone formation. In males under ADT, both testosterone and estrogen levels fall, shifting the balance of bone turnover toward resorption [48]. ADT does not have a significant impact on serum calcium, 25-hydroxyvitamin D, or PTH, but epidemiological studies have suggested that high levels of calcium intake may suppress PTH and ultimately 1,25-dihydroxyvitamin D and associated with increased risk of prostate cancer [49].

Current American Society of Clinical Oncology (ASCO) guidelines and expert panels suggest that patients under ADT with clinically significant bone loss should receive bisphosphonates, regardless of hormonal and metastatic status, and preclinical and clinical data show that bisphosphonates can also prevent and treat CTILB and may inhibit malignant bone disease development in patients with early stage disease [7,10].

## Conclusions

We conclude that patients with prostate cancer under androgen deprivation therapy had lower levels of BMD and higher rates of osteoporosis and fractures than patients with PCa not under ADT and healthy controls. Prostate cancer *per se *does not seem to be a risk factor for osteoporosis. However, the incidence of fractures was higher than that found in healthy controls, indicating that these patients may have had an additional, albeit unknown, mechanisms that could explain these findings. Although several studies in the literature have shown similar results, our study analyzed a larger number of studies and patients, providing consistent evidence on PCa, androgen deprivation therapy, osteoporosis and fracture risk.

## Competing interests

The authors declare that they have no competing interests.

## Authors' contributions

ASN - Conceived the study and participated in the design of the study, data collection, data extraction, statistical analysis and drafted the manuscript. Read and approved the manuscript. MTM - Conceived the study and participated in the design of the study and coordination. Read and approved the manuscript. MAPE - Participated in the data collection and drafted the manuscript. Read and approved the manuscript. MDS - Participated in the data collection and drafted the manuscript. Read and approved the manuscript. MLW - Conceived the study and participated in the design of the study and coordination. Read and approved the manuscript. FLAF - Conceived the study and participated in the design of the study and coordination. Read and approved the manuscript. RBR - Conceived the study and participated in the design of the study, data collection and coordination. Read and approved the manuscript. ACLP - Participated in the design of the study and coordination. Read and approved the manuscript. ADG - Conceived the study and participated in the design of the study and coordination. Read and approved the manuscript.

## Pre-publication history

The pre-publication history for this paper can be accessed here:

http://www.biomedcentral.com/1471-2490/10/9/prepub
